# Risk factors for upper urinary tract uroliths and ureteral obstruction in cats under referral veterinary care in the United Kingdom

**DOI:** 10.1111/jvim.16659

**Published:** 2023-03-01

**Authors:** Rebecca F. Geddes, Lucy J. Davison, Jonathan Elliott, Harriet M. Syme, Dan G. O'Neill

**Affiliations:** ^1^ Clinical Science and Services Royal Veterinary College Hawkshead Lane North Mymms Hatfield AL9 7TA United Kingdom; ^2^ Comparative Biological Sciences Royal Veterinary College, Royal College Street Camden London NW1 0TU United Kingdom; ^3^ Pathobiology and Population Science Royal Veterinary College Hawkshead Lane North Mymms Hatfield AL9 7TA United Kingdom

**Keywords:** cat, chronic kidney disease, nephrolithiasis, ureterolithiasis, urolith

## Abstract

**Background:**

Cats presenting with upper urinary tract uroliths (UUTUs) and ureteral obstruction (“obstructive UUTU”) are typically younger than cats with idiopathic CKD that often have incidental nephroliths.

**Hypothesis:**

Cats with upper urinary tract urolith have 2 clinical phenotypes; a more aggressive phenotype at risk of obstructive UUTU at a young age and a more benign phenotype in older cats, with reduced risk of obstructive UUTU.

**Objectives:**

Identify risk factors for UUTU and for obstructive UUTU.

**Animals:**

Eleven thousand four hundred thirty‐one cats were referred for care over 10 years; 521 (4.6%) with UUTU.

**Methods:**

Retrospective VetCompass observational cross‐sectional study. Multivariable logistic regression models were performed to identify risk factors for a diagnosis of UUTU vs no UUTU and additionally, obstructive UUTU vs nonobstructive UUTU.

**Results:**

Risk factors for UUTU included female sex (odds ratio [OR] 1.6, confidence interval [CI] 1.3‐1.9; *P* < .001), British shorthair, Burmese, Persian, Ragdoll or Tonkinese (vs non‐purebred ORs 1.92‐3.31; *P* < .001) breed and being ≥4 years (ORs 2.1‐3.9; *P* < .001). Risk factors for obstructive UUTU were female sex (OR 1.8, CI 1.2‐2.6; *P* = .002), having bilateral uroliths (OR 2.0, CI 1.4‐2.9; *P* = .002) and age, with the odds of obstructive UUTU increasing as age at diagnosis of UUTU decreased (≥12 years, reference category; 8‐11.9 years, OR 2.7, CI 1.6‐4.5; 4‐7.9 years, OR 4.1, CI 2.5‐7.0; 0‐3.9 years, OR 4.3, CI 2.2‐8.6; *P* < 0.001).

**Conclusions and Clinical Importance:**

Cats diagnosed with UUTU at a younger age have a more aggressive phenotype with higher risk of obstructive UUTU compared to cats over 12 years of age diagnosed with UUTU.

**Abbreviations**
ACEi: angiotensin converting enzyme inhibitor
AKI: acute kidney injury
ARB: angiotensin receptor blocker
BCS: body condition score
BW: bodyweight
CI: confidence interval
CKD: chronic kidney disease
CT: computed tomography
FLUTD: feline lower urinary tract disorder
HPF: high power field
IQR: interquartile range
IRIS: International Renal Interest Society
NSAIDs: nonsteroidal anti‐inflammatory drugs
QMHA: Queen Mother Hospital for Animals
RBCs: red blood cells
SBP: systolic blood pressure
SUB: subcutaneous ureteral bypass
UPCR: urine protein‐to‐creatinine ratio
UO: ureteral obstruction
USG: urine specific gravity
UUTU: upper urinary tract uroliths
WBCs: white blood cells

## 1 INTRODUCTION

Upper urinary tract uroliths (UUTU), affecting the kidney or ureter, can result in ureteral obstruction and acute kidney injury (AKI). Current guidelines advise placing a subcutaneous ureteral bypass (SUB) device or a ureteral stent to manage ureteral obstruction in cats,^1^ due to low success rates with medical management.^2^ Ureterolithiasis is the most common cause of ureteral obstruction in cats, recorded in 82% of obstructed ureters,^3^ or 96% of cats with ureteral obstruction undergoing SUB placement.^4^, ^5^ The frequency of UUTU increased 10‐fold, up to 68 per 10 000 cats, between 1981 and 2000 in 9 US veterinary teaching hospitals,^6^ and anecdotally this prevalence has continued to rise.

The majority of UUTU in cats are calcium oxalate (CaOx).^7^, ^8^ The preponderance of data on risk factors associated with CaOx urolith formation in cats stems from submissions to urolith analysis laboratories, but most submitted uroliths are from the lower urinary tract.^9^, ^10^, ^11^, ^12^, ^13^, ^14^, ^15^, ^16^ A contributory factor to this is that placement of a SUB does not require ureterolith removal and performing concurrent ureterotomy does not convey any recognized advantage to the cat.^17^ Factors associated with all‐cause ureteral obstruction,^18^ ureteral obstruction due to ureterolithiasis,^8^ and the presence of UUTU in cats with chronic kidney disease (CKD) are reported,^19^, ^20^ but an epidemiological study of all cats with UUTU is warranted.

Although all UUTU originate in the kidney, not all nephroliths migrate into the ureter. Risk factors for urolith migration in cats are underexplored and it is possible that cats at risk for ureterolithiasis and ureteral obstruction differ from cats with nonmigratory nephroliths. Fourteen cats with mean age 11.6 years, International Renal Interest Society (IRIS) stage 2‐3 CKD and concurrent nephrolithiasis did not develop subsequent ureteral obstruction,^21^ whereas this has been documented frequently in younger cats. The median age of 163 cats with ureteral obstruction due to ureterolithiasis was 7 years, with a wide age range from 8 months to 16 years.^8^ Additionally, 73% of cats diagnosed with CKD in a Japanese referral hospital had concurrent UUTU, with a median age of 5.6 years for the cats with urolithiasis, the majority of which (80%) had ureteroliths at the time of examination.^19^ These studies suggest a disparity in age between cats presenting with ureterolithiasis and cats with incidental nephroliths.

We hypothesized that 2 clinical phenotypes exist for cats with UUTU; a more aggressive phenotype at risk of ureteral obstruction at a young age and a more benign phenotype seen in older cats, with a lower risk of obstruction. The objectives of this study were first, to identify all cats diagnosed with UUTU over a 10‐year period at a UK tertiary referral hospital and to identify risk factors associated with a diagnosis of UUTU. The second objective was to identify risk factors for ureteral obstruction secondary to ureterolithiasis within those cats with UUTU.

## 2 MATERIALS AND METHODS

An observational cross‐sectional study design was used to identify the prevalence and risk factors associated with a diagnosis of UUTU within a cohort of cats seen at a UK veterinary referral hospital. Power calculations estimated 7626 cats were required to estimate prevalence for UUTU if occurring in 0.6% of cats with 0.1% acceptable margin of error at a 95% confidence level.^22^ Case records for all cats seen at the Queen Mother Hospital for Animals (QMHA) as a referral case at least once between 1 January 2009 and 1 January 2019 were searched within the VetCompass Programme. Ethical approval was granted by the Royal Veterinary College Clinical Research Ethical Review Board (SR20181652).

To be included as a “UUTU case,” a cat had to have a diagnosis of UUTU made during a hospital visit based on the presence of at least 1 urolith in a kidney or ureter on abdominal imaging (ultrasound, radiographs, or computed tomography [CT]) performed or reviewed by a board‐certified specialist in small animal diagnostic imaging. Search terms were explored individually for their ability to identify candidate (ie, possible) cases with a high probability of having UUTU. The final list of search terms used on 21 August 2020 to search clinical notes from 1 January 2009 to 21 August 2020 is shown in Supporting Information. Clinical records for all candidate cases were reviewed by a board‐certified specialist in internal medicine (RG) to confirm cats that met the criteria for being a UUTU case.

Data were extracted for confirmed cases as shown in Supporting Information. In brief, these data included: signalment, reported reason for referral, arrival date of visit when uroliths were first identified at the QMHA (baseline), history including a previous diagnosis of CKD, diet fed and indoor/outdoor status, clinical signs at presentation, physical examination, imaging findings, biochemical variables, ionized calcium, urinalysis and culture, and follow‐up information where available. For data fields with repeated measurements, the earliest values at or after the baseline visit were extracted for each case. Results of urolith analysis from any timepoint were extracted. Cats that were reviewed as candidate UUTU cases but did not have uroliths present in their kidneys or ureters and cats that were not flagged as candidate cases were categorized as “non‐cases.”

Only biochemical data from the QMHA Diagnostic Laboratory were included in analyses to ensure consistency. Ionized calcium was measured on 3 different point‐of‐care analyzers during the 10‐year study period: NOVA biomedical (CCX Blood Gas s/n Y0240301Z; January 2009‐September 2016), Radiometer ABL800 Flex Analyzer (October 2016‐study end) and iSTAT 1 (Abaxis VetScan 300V, s/n 705971; periodic use May 2017‐study end). Based on clinical experience and calculation of an in‐house 95% reference interval for the Radiometer ABL800 using healthy blood donors, ionized hypercalcemia was defined as values >1.41 mmol/L.^23^

A diagnosis of ureteral obstruction relied on statements to this effect made by board‐certified diagnostic imaging specialists in the case records and included cats considered to have partial or complete obstruction. “Big kidney little kidney” syndrome was defined as a difference in longitudinal kidney measurements on imaging of >0.7 cm between the contralateral kidneys.^24^

For UUTU cases, age was recorded at the baseline visit (when first diagnosed) and for non‐cases, age was recorded on the final visit date to the QMHA before or on 1 January 2019, whichever came first to allow as much time as possible to be diagnosed with UUTU before classification as a non‐case. Neuter status was excluded from analyses because “entire” status was the default option within the hospital's computer software, therefore missing selection of this box during cat data entry might have led to cats being misclassified regarding neuter status. Additionally, the system did not offer an option to record the date of neutering, therefore neuter status at the time of UUTU diagnosis could not be confirmed.

Statistical analysis was performed using R (R ×64 4.1.2). Categorical data were reported as proportions. Continuous data were assessed for normality using histograms, Q‐Q plots, and the Shapiro Wilk test. Data were reported as median (interquartile range [IQR]) or mean (±SD) as appropriate. Comparisons between groups were made using *t* tests or Wilcoxon rank sum tests for continuous, and chi‐squared analyses for categorical data. Two multivariable logistic regression models were built to identify independent risk factors after accounting for the effects of other variables. The first model identified risk factors associated with a diagnosis of UUTU by examining the association of signalment variables (sex, breed, and age) with being a UUTU case (outcome 1) or non‐case (outcome 0). Breed descriptive information was cleaned and mapped to a VetCompass breed list derived and extended from the VeNom Coding breed list.^25^ The breed type variable included (i) individual breeds with a minimum of 3 UUTU cases or 100 non‐cases in the overall study sample, (ii) a grouped category for all non‐purebred cats (domestic shorthair, domestic mediumhair, domestic longhair, and cases stated to be crossbred without a specified breed), and (iii) a grouped category for all other purebred and purebred‐crosses. Continuous variables were assessed for linearity against the dependent variable and were converted into categorical variables if found to be nonlinearly associated. In consequence, age was categorized as 4 categories: 0‐3.9 years, 4.0‐7.9 years, 8.0‐11.9 years, and ≥12 years.

The second logistic regression model identified risk factors associated with ureteral obstruction due to calcium‐based ureterolithiasis. Cats with ureteroliths known or suspected not to be calcium‐based (eg, due to confirmation of a different urolith composition on analysis at any timepoint or due to a diagnosis of a congenital portosystemic shunt) were excluded from this analysis. Cats documented to have ureteral obstruction due to causes other than ureterolithiasis were also excluded. Cats suspected to have calcium‐based UUTU were categorized into 2 groups: (i) cats documented to have ureterolithiasis and a partial or complete ureteral obstruction at any timepoint were categorized as “obstructive UUTU” (outcome 1) or (ii) cats never documented to have ureteral obstruction were categorized as “nonobstructive UUTU” (outcome 0). Variables assessed for association with obstructive UUTU were those not directly influenced by the presence of ureteral obstruction (sex, age, bodyweight, purebred vs non‐purebred, previous CKD diagnosis, fed dry food only vs mixed or wet food, indoor/outdoor vs indoor only, bilateral vs unilateral uroliths). Age was included as a categorical variable with 4 categories: 0‐3.9 years, 4.0‐7.9 years, 8.0‐11.9 years, and ≥12 years.

For both logistic regression models, variables significant at *P* < .2 in univariable analyses were carried forwards into backwards, stepwise multivariable logistic regression model‐building. A category termed “not reported” was created to allow inclusion within variables with data missing for >10 individuals in the multivariable models. Model assumptions were checked by assessing correlations between independent variables, linearity of continuous variables against the log odds of the dependent variable and the presence of outliers using Cooks Distance values.

## 3 RESULTS

The total cohort seen at least once as referral cases at the QMHA between 1 January 2009 and 1 January 2019 included 11 431 cats. From the total cohort of 11 431, 912 cats were identified as candidate cases and 521 were confirmed to have UUTU, therefore, the overall prevalence of UUTU diagnosis during the 10‐year period was 4.6% (95% CI 4.2‐5.0). The prevalence of nephrolithiasis was 3.8% (95% CI 3.5‐4.2) and the prevalence of ureterolithiasis was 2.4% (95% CI 2.2‐2.7; 193 cats [1.7%] had both). The annual case count for a diagnosis of UUTU was: 2009 n = 14, 2010 n = 18, 2011 n = 31, 2012 n = 23, 2013 n = 34, 2014 n = 75, 2015 n = 76, 2016 n = 83, 2017 n = 83, 2018 n = 84. Two thirds of the UUTU cases, 344/521 (66.0%) had been referred from external primary care practices for investigations into urolithiasis or azotemia. An additional 9/521 (1.7%) UUTU cases were initially seen by the First Opinion Out of Hours Service at QMHA with subsequent internal referral. The remaining 168/521 (32.2%) UUTU cases were referred for other reasons with incidental documentation of uroliths during clinical investigations.

### 3.1 Risk factors associated with a diagnosis of UUTU

Upper urinary tract urolith cases were older than non‐cases (median 8.0 [IQR 5.3‐11.3] years vs 7.4 [3.2‐11.9] years; *P* < .001) and were more likely to be female (54.1% female vs non‐cases 43.4% female; *P* < .001). Signalment data for the 521 UUTU cases and 10 910 non‐cases are summarized in Table 1. Multivariable logistic regression identified 3 independent risk factors associated with diagnosis of UUTU: being female, being over 4 years of age and being 1 of 5 specific pure‐breeds when compared to non‐purebred cats: Ragdoll, Tonkinese, Persian, British shorthair, and Burmese. The Norwegian Forest was at reduced risk of UUTU compared to non‐purebred cats (OR 0.11, 95% CI 0.01‐0.47). The age category with the highest odds of a diagnosis of UUTU was 4‐7.9 years (OR 3.9, 95% CI 2.94‐5.25) when compared to the baseline category of <4 years (see Table 2).

**TABLE 1:** Signalment data for 521 cats with upper urinary tract uroliths (UUTU cases) and 10 910 cats with no diagnosis of upper urinary tract uroliths (non‐cases) seen at a UK referral hospital over 10 years from 1 January 2009 to 1 January 2019.

	UUTU cases	Non‐cases
	*n* (%)	*n* (%)
Sex	Total n = 521	Total n = 10 896
Male	239 (45.9)	6170 (56.6)
Female	282 (54.1)	4726 (43.4)
Neuter status	Total n = 521	Total n = 10 910
Neutered	490 (94.0)	9610 (88.1)
Entire	31 (6.0)	1300 (11.9)
Breed	Total n = 521	Total n = 10 910
Domestic shorthair	281 (53.9)	6605 (60.5)
Domestic longhair	43 (8.3)	789 (7.2)
British shorthair	40 (7.7)	473 (4.3)
Persian	24 (4.6)	215 (2.0)
Ragdoll	19 (3.6)	168 (1.5)
Burmese	18 (3.5)	215 (1.0)
Bengal	13 (2.5)	302 (2.8)
Siamese	12 (2.3)	227 (2.1)
Maine Coon	10 (1.9)	237 (2.2)
Birman	7 (1.3)	232 (2.1)
Domestic mediumhair	7 (1.3)	192 (1.8)
Crossbreed—no breed specified	6 (1.2)	155 (1.4)
British Blue	6 (1.2)	122 (1.1)
Tonkinese	6 (1.2)	45 (0.4)
Russian Blue	5 (1.0)	69 (0.6)
Exotic shorthair	4 (0.8)	54 (0.5)
Oriental shorthair	3 (0.6)	35 (0.3)
Crossbreed—breed specified	2 (0.4)	116 (1.1)
Devon Rex	2 (0.4)	26 (0.2)
Exotic longhair	2 (0.4)	6 (0.1)
Norwegian Forest	1 (0.2)	217 (2.0)
Siberian	1 (0.2)	46 (0.4)
European shorthair	1 (0.2)	20 (0.2)
Scottish Fold	1 (0.2)	16 (0.1)
Korat	1 (0.2)	11 (0.1)
Havana Brown	1 (0.2)	7 (0.1)
Manx	1 (0.2)	7 (0.1)
American shorthair	1 (0.2)	6 (0.1)
Selkirk Rex	1 (0.2)	5 (0.05)
Snowshoe	1 (0.2)	4 (0.04)
Ragamuffin	1 (0.2)	2 (0.02)
Other purebreds	0 (0)	267 (2.4)
Age (years)	8.0 (5.3‐11.3)	7.4 (3.2‐11.9)

**TABLE 2:** Logistic regression modeling to identify risk factors for a diagnosis of upper urinary tract uroliths within 11 431 cats seen at a UK referral hospital over 10 years from 1 January 2009 to 1 January 2019.

Univariable models				
Variable	Category	OR	95% CI	*P* value
Sex	Male	BASE		<.001
	Female	1.56	1.30‐1.86	
Breed	Non‐pedigree	BASE		.71
	Pedigree (all breeds)	0.93	0.65‐1.34	
Breed	Non‐pedigree	BASE		<.001
	Bengal	0.98	0.53‐1.66	
	Birman	0.69	0.29‐1.36	
	British Blue	1.13	0.44‐2.37	
	British shorthair	1.93	1.35‐2.68	
	Burmese	1.91	1.13‐3.04	
	Maine Coon	0.87	0.41‐1.61	
	Norwegian Forest	0.11	0.01‐0.47	
	Oriental shorthair	1.95	0.47‐5.46	
	Persian	2.54	1.61‐3.86	
	Ragdoll	2.59	1.55‐4.11	
	Russian Blue	1.65	0.58‐3.73	
	Siamese	1.21	0.63‐2.08	
	Tonkinese	3.11	1.18‐6.80	
	Purebred other	0.76	0.46‐1.17	
Age (years)	0‐3.9	BASE		<.001
	4.0‐7.9	3.79	2.87‐5.07	
	8.0‐11.9	3.03	2.26‐4.09	
	≥12	2.03	1.46‐2.78	

Multivariable model				
Variable	Category	OR	95% CI	*P* value
Sex	Male	BASE		<.001
	Female	1.60	1.34‐1.92	
Breed	Non‐pedigree	BASE		<.001
	British shorthair	2.02	1.41‐2.81	
	Burmese	1.92	1.13‐3.07	
	Norwegian Forest	0.11	0.01‐0.47	
	Persian	2.59	1.62‐3.95	
	Ragdoll	3.31	1.95‐5.34	
	Tonkinese	3.15	1.18‐7.02	
Age (years)	0‐3.9	BASE		<.001
	4.0‐7.9	3.9	2.94‐5.25	
	8.0‐11.9	3.13	2.33‐4.25	
	≥12	2.10	1.53‐2.89	

Abbreviations: OR, odds ratio; 95% CI, 95% confidence interval of the odds ratio.

### 3.2 Historical data for cases diagnosed with UUTU

Data regarding diagnoses made before referral, medications administered before referral, diet being fed at the time of referral, cat lifestyle, and presenting clinical signs are detailed in Table 3. Eighty‐eight (16.9%) of UUTU cases had a previous diagnosis of CKD. In 19/88 (21.6%), CKD was documented to precede the diagnosis of uroliths, in 25/88 (28.4%) uroliths were documented to precede the diagnosis of CKD, and it was unclear in the records which came first in 44/88 (50.0%) cats. Of note, 3 cases had been diagnosed with a congenital portosystemic shunt and 1 cat had a history of hypervitaminosis D.

**TABLE 3:** Historical data for cases diagnosed with upper urinary tract uroliths (UUTU) (n = 521).

Data	Sample n	Number of UUTU cases (%)
Previous diagnoses	521	
No history of previous important illness		271 (52.0%)
CKD		88 (16.9%)
FLUTD		52 (10.0%)
Dental disease		25 (4.8%)
Chronic enteropathy		21 (4.0%)
Neoplasia		20 (3.8%)
UTI		18 (3.5%)
Previous AKI		15 (2.9%)
Diabetes mellitus		15 (2.9%)
Respiratory disease		14 (2.7%)
Hyperthyroidism		13 (2.5%)
Osteoarthritis		13 (2.5%)
Medication administered for >1 month before referral	521	
None		421 (80.8%)
Antibiotics		25 (4.8%)
ACEi/ARBs		25 (4.8%)
Insulin		13 (2.5%)
NSAIDs		12 (2.3%)
Medications administered within 1 month of referral	521	
Fluid therapy administered IV		166 (31.9%)
Antibiotics		150 (28.8%)
None		140 (26.9%)
Opioids		84 (16.1%)
NSAIDs		48 (9.2%)
Antiemetics		44 (8.4%)
Gastroprotectants		41 (7.9%)
ACEi/ARBs		35 (6.7%)
Diet consistency fed at the time of referral	175	
Dry food only		86 (49.1%)
Canned and dry food		61 (34.9%)
Canned food only		28 (16.0%)
Diet category fed at the time of referral	521	
Life‐stage appropriate/no specific information recorded		370 (71.0%)
Renal diet		77 (14.8%)
Urinary diet		36 (6.9%)
Another prescription diet		37 (7.1%)
Chicken/fish only		1 (0.002%)
Lifestyle at time of referral	267	
Indoor/outdoor		154 (57.7%)
Indoor only		113 (42.3%)
Outdoor only		0
Presenting clinical signs	521	
Hypo/anorexia		240 (46.0%)
Lethargy		177 (34.0%)
Vomiting		155 (29.8%)
Weight loss		114 (21.9%)
Polyuria/polydipsia		86 (16.5%)
Signs of lower urinary tract disease		64 (12.3%)
Reported to be clinically normal		33 (6.3%)

Abbreviations: ACEi, angiotensin converting enzyme inhibitor; AKI, acute kidney injury; ARB, angiotensin receptor blockers; CKD, chronic kidney disease; FLUTD, feline lower urinary tract disorders; NSAIDs, nonsteroidal anti‐inflammatory drugs; UTI, urinary tract infection.

### 3.3 Physical examination and imaging findings in cases diagnosed with UUTU

Abnormalities were not detected on physical examination at baseline in 106/521 (20.3%) UUTU cases. Abdominal palpation revealed enlarged kidney(s) in 146 (28.0%), small kidney(s) in 102 (19.6%), painful kidney(s) in 72 (13.8%), and irregular kidney(s) in 33 (6.3%) cats. Pyrexia (rectal temperature >39.5°C) was noted in 8 (1.5%) cats, and 8 (1.5%) cats were dehydrated on examination. Bodyweight, body condition score (BCS), blood pressure, and other baseline clinicopathologic data for all UUTU cases are reported in Table 4.

**TABLE 4:** Baseline data for 521 cats with a diagnosis of upper urinary tract uroliths.

Variable	Sample n	Median (IQR) or n (%)
Bodyweight (kg)	460	3.91 (3.19‐4.86)
Body condition score (out of 9)	328	4 (3‐5)
SBP (mm Hg)	159	138 (120‐160)
Creatinine (mg/dL)	415	2.6 (1.6‐6.2)
Urea (mg/dL)	414	47.9 (30.5‐114.0)
Total calcium (mg/dL)	412	9.8 (9.2‐10.5)
Phosphorus (mg/dL)	413	5.5 (4.3‐8.0)
Potassium (mEq/L)	412	4.5 (4.1‐5.0)
PCV (%)	459	31 (25‐35)
TS (g/dL)	468	7.2 (6.5‐7.9)
USG	375	1.018 (1.014‐1.029)
UPCR	75	0.41 (0.19‐1.00)
Urine pH	367	6 (5.5‐6.5)
Crystals present on sediment	378	46 (12.2%)
Calcium oxalate		23
Struvite		7
Other		16
Casts present on sediment	378	45 (11.9%)
Granular		23
Hyaline		15
Other		7
Pyuria (>5 WBCs per HPF)	364	49 (13.5%)
Microscopic hematuria (>10 RBCs per HPF)	364	160 (44.0%)
Gross hematuria	368	29 (7.9%)
Bacteriuria present on sediment	382	38 (9.9%)
Positive urine culture from bladder	375	60 (16.0%)
*E. coli*		35
*Enterococcus*		15
*Staphylococcus*		4
*Pseudomonas*		1
*Bacillus* spp.		1
*Enterobacter cloacae*		1
*E. coli* and *Enterococcus*		1
*E. coli* and *Staphylococcus*		1
*Enterococcus*, *Staphylococcus* and *Streptococcus*		1
Urine culture from pyelocentesis	81	5 (6.2%)
*E. coli*		1^a^
*Enterococcus*		2^a^ ^,^ ^b^
*Staphylococcus*		2^b^ ^,^ ^c^

Abbreviations: HPF, high power field; RBCs, red blood cells; SBP, systolic blood pressure; TS, total solids; UPCR, urine protein‐to‐creatinine ratio; USG, urine specific gravity; WBCs, white blood cells.

^a^ Previous urine culture from cystocentesis samples negative.

^b^ Previous culture from cystocentesis sample positive for *Enterococcus*.

^c^ Previous culture from cystocentesis samples positive for *Staphylococcus*.

Imaging during the baseline visit for UUTU cases included abdominal ultrasound in 442/521 (84.8%), radiographs in 78/521 (15.0%), and CT scans in 68/521 (13.1%). Of the UUTU cases, 439/521 (84.3%) had nephroliths present with the following distribution: bilateral in 234 (53.3%), right kidney only in 91 (20.7%), left kidney only in 114 (26.0%). Ureteroliths were present in 277/521 (53.2%) UUTU cases: bilateral in 87 cats (31.4%), right kidney only in 78 (28.2%), and left kidney only in 112 (40.4%). Cystoliths were documented in 109/521 (20.9%) UUTU cases and an additional 86/521 (16.5%) had “hyperechoic sediment” in the urinary bladder. Urethral calculi were documented in 16/521 (3.1%). Overall, uroliths within the upper urinary tract were present bilaterally in 308/521 (59.1%), unilaterally on the right in 95/521 (18.2%), and unilaterally on the left in 118/521 (22.6%) of UUTU cases. Nephrocalcinosis/parenchymal mineralization was reported in 95/521 (18.2%) of UUTU cases.

Five UUTU cases had only 1 kidney present. “Big kidney little kidney” syndrome was documented in 171/516 (33.1%) remaining UUTU cases. Severe hydronephrosis with only a thin rim of kidney parenchyma visible was present in 24 UUTU cases, affecting the left kidney in 12 (50%) cats and affecting the right kidney in another 12 (50%) cats. During their baseline visit, 283/521 (54.3%) UUTU cases were considered to have at least a unilateral ureteral obstruction, which was secondary to ureterolithiasis in 276/283 (97.5%) cases. Fifty‐seven UUTU cases had an anterior pyelogram performed to confirm the presence of ureteral obstruction and in 8/57 cases, the ureter was subsequently considered not to be obstructed due to visualization of contrast down the entire length of the ureter. Initial management strategies for the remaining 275 UUTU cases with ureteral obstruction (of any cause) were: SUB placement 107/275 (38.9%), of which 70 were unilateral and 37 were bilateral; medical management only 98/275 (35.6%); euthanasia 35/275 (12.7%); stent placement 24/275 (8.7%), of which 21 were unilateral and 3 were bilateral; ureterotomy 5/275 (1.8%); nephrectomy 4/275 (1.5%); cystotomy 1/275 (0.4%) and referred to another hospital 1/275 (0.4%). Four of the 238 UUTU cases without ureteral obstruction underwent cystotomy to remove cystoliths.

### 3.4 Additional clinical detail and outcomes for UUTU cases

The most common diagnoses in UUTU cases made during the baseline visit included AKI (291/521; 55.6%), CKD (271/521; 52.0%), neoplasia (58/521; 11.1%), pyelonephritis (32/521; 6.1%), urinary tract infection (28/521; 5.4%), systemic hypertension (22/521; 4.2%), cardiomyopathy (18/521; 3.5%), and FLUTD (17/521; 3.3%). Ionized calcium was measured in 359 UUTU cases, of which 62/359 (17.3%) were documented to have ionized hypercalcemia (>1.41 mmol/L). The diagnoses reached for hypercalcemia were: no final diagnosis reached 26/62 (41.9%), transient 16/62 (25.8%), idiopathic hypercalcemia 10/62 (16.1%), associated with CKD or feeding a clinical renal diet 7/62 (11.3%), young age 1/62 (1.6%), neoplasia 1/62 (1.6%), and granulomatous disease 1/62 (1.6%). Total hypercalcemia (>11.6 mg/dL, the top of the laboratory reference interval) was documented in 47/416 (11.2%) cats, of which 20/33 had concurrent ionized hypercalcemia.

Urine was obtained from 414 UUTU cases at the baseline visit but not every sample had a complete urinalysis and culture performed. Urine specific gravity (USG), urine protein‐to‐creatinine ratio (UPCR), and sediment analysis data are shown in Table 4. Urolith analysis had been performed on uroliths retrieved from 46 UUTU cases (uroliths removed from ureter in 20 cats, bladder in 23 cats, and both in 3 cats), and all uroliths were confirmed to be majority CaOx except from 1 cat with xanthine uroliths. In 4/521 (0.8%) UUTU cases, uroliths were suspected not to be CaOx; the cat with xanthine uroliths and 3 cats with untreated congenital portosystemic shunts all <9 months of age. In total, 421/521 (80.8%) UUTU cases survived to discharge from the hospital. Median follow‐up time for all 521 UUTU cases was 33 days (range, 0‐2581 days). At last follow‐up, 311/521 (59.7%) UUTU cases were still alive and 210/521 (40.3%) were dead. Of the dead cats, urolithiasis was cited as the likely cause of death or reason for a decision of euthanasia in 121/210 (57.6%) cases.

### 3.5 Risk factors associated with obstructive UUTU

Of the 517 cases with confirmed or suspected calcium‐based UUTU, 290 (56.1%) cats were documented to have at least a partial ureteral obstruction due to ureterolithiasis and therefore were categorized as “obstructive UUTU.” In the majority of the obstructive UUTU cases, the obstruction was documented at the cat's baseline hospital visit (275/290 [94.8%]). The remaining 227 UUTU cases were categorized as “nonobstructive UUTU”; including 6 cats with ureteroliths documented but without any evidence of ureteral obstruction present (no ureteral dilation or renal pelvic dilation noted). However, 4/227 nonobstructive UUTU cases were documented to have ureteral obstruction due to other causes (ureteritis [n = 2] or neoplasia [n = 2]) and were therefore removed from subsequent analyses. Cats with obstructive UUTU (n = 223) were significantly younger (7.0 [4.8‐9.9] years vs nonobstructive UUTU cases 10.0 (6.7‐13.0) years; *P* < .001) and a greater proportion of obstructive UUTU cases had ionized hypercalcemia (48/229 [21.0%] vs nonobstructive UUTU cases 14/127 [11.0%]; *P* < .001). Additional baseline data for cats with obstructive and nonobstructive UUTU are shown in Table 5. Within UUTU cases, female cats had significantly lower bodyweights (3.47 [2.90‐4.24] kg vs male cats 4.55 [3.79‐9.70] kg; *P* < .001). There was no difference in the number of cats exclusively fed dry food between UUTU cats with bilateral vs unilateral uroliths (bilateral uroliths 57/106 [53.8%] vs unilateral uroliths 29/69 [42.0%]; *P* = .17). Logistic regression modeling identified being of younger age, being female and having bilateral uroliths as independent risk factors for obstructive UUTU, see Table 6. The younger the age category at diagnosis of UUTU, the higher the odds were for a cat to be diagnosed with obstructive UUTU. Cats 0‐3.9 years of age demonstrated 4.3 times (95% CI 2.19‐8.71) the odds for obstructive UUTU compared to cats over 12 years of age, see Table 6.

**TABLE 5:** Baseline data for cats suspected or confirmed to have calcium‐based uroliths, and with documentation of ureteral obstruction due to ureterolithiasis (obstructive UUTU; n = 290) compared to cats with UUTU and no evidence of ureteral obstruction (n = 223).

	Nonobstructive UUTU	Obstructive UUTU	*P* value
	n (%) or median (IQR)	n (%) or median (IQR)	
Female	102/223 (45.7%)	177/290 (61.0%)	<.001
Pedigree/pedigree cross	84/223 (37.7%)	104/290 (36.9%)	.74
History of CKD diagnosis	35/223 (15.7%)	51/290 (17.6%)	.65
Diagnosed with CKD at baseline	87/223 (39.0%)	181/290 (62.4%)	<.001
Fed dry food only	19/69 (27.5%)	67/105 (63.8%)	<.001
Indoor only cat	43/108 (39.4%)	68/156 (43.6%)	.63
Referred for stone disease	74/223 (33.2%)	265/290 (91.4%)	<.001
Age (years)	10.0 (6.7‐13.0)	7.0 (4.8‐9.8)	<.001
Bodyweight (kg)	4.1 (3.4‐5.2)	3.8 (3.1‐4.7)	.005
Body condition score (out of 9)	4 (3‐5)	4 (3‐5)	.17
SBP (mm Hg)	140 (120‐160)	138 (120‐155)	.52
Creatinine (mg/dL)	1.7 (1.3‐2.5)	4.4 (2.4‐10.8)	<.001
Urea (mg/dL)	32.8 (23.8‐47.9)	76.2 (41.3‐162.6)	<.001
Total calcium (mg/dL)	9.6 (8.7‐10.4)	9.9 (9.4‐10.7)	<.001
Ionized calcium (mmol/L)	1.30 (1.10‐1.40)	1.37 (1.18‐1.47)	.03
Ionized hypercalcemia	14/127 (11.0%)	48/229 (21.0%)	.03
Phosphate (mg/dL)	4.6 (4.0‐5.8)	6.4 (4.8‐11.8)	<.001
Potassium (mEq/L)	4.4 (4.0‐4.8)	4.7 (4.2‐5.3)	<.001
PCV (%)	32 (25‐36)	30 (26‐35)	.57
Total solids (g/dL)	7.1 (6.4‐7.7)	7.2 (6.6‐8.0)	.03
USG	1.024 (1.016‐1.036)	1.015 (1.012‐1.022)	<.001
Urine pH	6.0 (5.5‐6.5)	6.0 (5.5‐6.0)	<.001
UPCR	0.37 (0.16‐0.91)	0.56 (0.33‐1.00)	.17
Positive urine culture	20/131 (15.3%)	42/236 (17.8%)	.64
Bilateral stones	112/223 (50.2%)	193/290 (66.6%)	<.001

Abbreviations: CKD, chronic kidney disease; UPCR, urine protein‐to‐creatinine ratio; USG, urine specific gravity; SBP, systolic blood pressure.

**TABLE 6:** Logistic regression modeling to identify risk factors for obstructive upper urinary tract uroliths within 513 cats with a diagnosis of upper urinary tract uroliths presumed or confirmed to be calcium based.

Univariable models				
Variable		OR	95% CI	*P* value
Female		1.86	1.31‐2.65	<.001
Age (years)	≥12	BASE		<.001
	8‐11.9	2.69	1.62‐4.53	
	4‐7.9	4.53	2.76‐7.57	
	0‐3.9	4.74	2.45‐9.46	
Pedigree or pedigree cross		.93	.64‐1.33	.67
Previous CKD diagnosis		1.15	.72‐1.85	.57
Fed dry food only		4.64	2.32‐9.15	<.001
Indoor/outdoor vs indoor		.54	.52‐1.41	.54
Bilateral vs unilateral stones		1.97	1.38‐2.83	.001
Bodyweight (kg)		.82	.70‐.95	.007

Final multivariable model				
Variable		OR	95% CI	*P* value
Age (years)	≥12	BASE		<.001
	8‐11.9	2.66	1.58‐4.54	
	4‐7.9	4.15	2.51‐7.01	
	0‐3.9	4.27	2.17‐8.64	
Female		1.80	1.24‐2.62	.004
Bilateral vs unilateral stones		1.97	1.35‐2.89	<.001

Abbreviations: CI, confidence interval; CKD, chronic kidney disease; OR, odds ratio.

## 4 DISCUSSION

This study reports a high prevalence (4.6%) of cats referred for assessment diagnosed with UUTU during a 10‐year period in a UK referral hospital. Being a pure‐bred cat per se did not increase the risk of a UUTU diagnosis, however, a number of specific purebreds had an increased risk for UUTU compared to non‐purebred cats. Independent risk factors for UUTU diagnosis included being female, being British shorthair, Burmese, Persian, Ragdoll or Tonkinese, and being over 4 years of age, with cats of 4.0‐7.9 years most at risk. Cats diagnosed with obstructive UUTU were younger than cats that had nonobstructive uroliths, and a higher proportion were eating dry food only or demonstrated ionized hypercalcemia. Independent risk factors for a UUTU case having obstructive UUTU were being under 12 years of age, being female, and having uroliths present bilaterally within the urinary tract.

Upper urinary tract uroliths were noted with a prevalence of 4.6%, whereas a previous study from 9 US teaching hospitals in 1999, reported a prevalence of 0.68%.^6^ Notably, this prevalence will likely to be an underestimate because not all cats seen at the QMHA during the 10‐year period underwent abdominal imaging, and therefore, some cases with UUTU will have been missed. A diagnosis of UUTU was associated with poor outcomes for many cats in the present study, with 19.2% of UUTU cases not surviving to discharge and 40.3% of UUTU cases deceased by last follow‐up time of (median 33 days), with urolithiasis cited as the cause of death or reason for euthanasia in 57.6%. These data emphasize the importance of UUTU and highlight the need for further studies into the prevention of UUTU formation in cats.

Sixty‐five percent of cats diagnosed with UUTU were non‐purebred cats and taken as a combined group, purebred cats did not have an increased risk of a UUTU diagnosis compared to non‐purebred cats. However, 5 specific purebreds (British shorthair, Persian, Burmese, Ragdoll, and Tonkinese) were at increased odds for a diagnosis of UUTU compared to non‐purebred cats. Tonkinese cats are at risk for renal calculi.^7^ Additionally, Burmese,^10^, ^15^ Persian,^10^, ^15^ and Tonkinese^10^ cats are predisposed to forming CaOx uroliths compared to DSH cats. Norwegian Forest cats had lower odds for a diagnosis of UUTU, with only 1 of 218 receiving this diagnosis during the study period. Incidental uroliths could have been missed as individuals of this breed were invited to the QMHA for echocardiography during the period evaluated^26^ and did not routinely undergo abdominal imaging. However, Norwegian Forest cats are underrepresented in previous studies of nephrolithiasis^7^ and ureterolithiasis^8^ and in urolith datasets,^10^, ^12^, ^15^ therefore this breed might be of interest for future studies as a breed with possible protection from the development of uroliths.

Female cats had 1.6 times the odds for a diagnosis of UUTU compared to male cats. Additionally, within cats with urolithiasis, female cats had increased odds for developing obstructive UUTU. A previous study of 163 cats with ureterolithiasis reported a higher proportion of female cats (57.7%),^8^ whilst renal calculi have not been found to be associated with either sex.^7^ Male cats have increased risk for CaOx urolithiasis,^10^, ^12^, ^15^, ^16^, ^27^ but those data are mostly based on urolith submissions from the lower urinary tract, which might be biased by the difference in male vs female anatomy and an increased likelihood for male cats to develop urethral obstruction necessitating intervention and urolith removal. If female cats are at increased risk for developing UUTU, this contrasts with human patients where nephroliths are more prevalent in men.^28^, ^29^ However, recent studies reported that male‐to‐female ratios for human kidney urolith diagnoses have declined markedly, largely due to an increase in urolithiasis in women.^29^, ^30^ An important difference when comparing these species is that the majority of cats are neutered, therefore the protective effect of estrogen on nephrolith formation seen in women will not apply,^31^ however, this is insufficient to explain the disparity in risk of ureteral obstruction between neutered female and male cats. One potential theory is the difference in bodyweight (and therefore size) between female and male cats, with both mixed‐breed and purebred female cats frequently demonstrating mean adult bodyweights that are 0.7 to >1.0 kg lower than their male counterparts.^32^, ^33^ In the present study, the median bodyweight for female UUTU cases was significantly lower (by just over 1 kg) than for the male UUTU cases and bodyweight was a significant risk factor for obstructive UUTU in univariable analyses, but did not remain in the multivariable model, possibly due to the association between sex and bodyweight. Further work is required to confirm if a smaller bodyweight/size and therefore potentially narrower ureters could increase the risk for urolithiasis‐associated ureteral obstruction in female cats.

Our data support the study hypothesis that there are 2 clinical phenotypes of cats that have upper urinary tract urolithiasis; a more aggressive phenotype at risk of ureteral obstruction at a younger age and a more benign phenotype seen in older cats, with a lower risk of ureteral obstruction. Cats that developed obstructive UUTU were significantly younger (median 7.0 years) than cats with nonobstructive uroliths (median 10.0 years). Additionally, age was an independent risk factor for obstructive UUTU, with the odds of a cat developing ureteral obstruction increasing as the age at UUTU diagnosis decreased. Cats diagnosed with UUTU at 0‐3.9 or 4‐7.9 years of age had more than 4 times the odds for a diagnosis of obstructive UUTU compared to cats over 12 years of age. Having upper urinary uroliths bilaterally was also an independent risk factor for obstructive UUTU, suggesting that cats with a higher propensity to form nephroliths are also at higher risk for migration of nephroliths into their ureters, in accordance with the hypothesis of a more aggressive phenotype in younger cats. Interestingly, a higher proportion of cats with obstructive UUTU were exclusively fed dry food, which is a risk factor for development of UUTU in cats with CKD,^19^ and is a risk factor for ureteral obstruction (all causes) in cats,^18^ however, this was not found to be an independent risk factor for obstructive UUTU in the multivariable model.

The etiological relationship between CKD and UUTU in cats has long been queried: is kidney disease a cause or consequence of urolith formation?^11^ A high proportion of cats diagnosed with CKD have UUTU.^19^, ^20^ Additionally, over 75% of cats with CKD demonstrate microscopic mineralization of their kidneys at postmortem,^34^ and it is unclear whether medullary mineralization is on a continuum with nephrolith formation. However, in the present study, over half of UUTU cases (52%) had no record of previous illnesses, 80.8% were not receiving long‐term medications, and only 19/521 (3.6%) had been diagnosed with CKD before uroliths were documented. During their baseline visit, 51.8% of cats with UUTU were concurrently diagnosed with CKD, but this proportion was significantly higher for cats diagnosed with obstructive UUTU (62.4%) vs nonobstructive uroliths (39.2%). Additionally, a third of UUTU cases had “big kidney little kidney” syndrome, suggesting prior ureteral obstruction might have occurred undocumented in a proportion of cases, and highlighting the potential consequence of ureteral obstruction on kidney size and function. Taken together, these data suggest that in most cats with UUTU, the nephroliths form first and this increases the risk for subsequently developing CKD, as is the case in human patients.^35^, ^36^ In agreement with this, a retrospective case‐control study found that 65.9% of cats with nephroliths or ureteroliths were diagnosed with CKD compared to only 30% of controls.^37^ Prospective longitudinal studies with repeated urinary tract imaging and kidney function assessment would be required to confirm whether UUTU development precedes the development of CKD in cats.

In this study, assumptions were made that UUTU cases had calcium‐containing uroliths, unless there was evidence to the contrary. This was based on studies that have consistently found that the most common UUTU from cats are calcium‐based and in particular CaOx,^7^, ^8^, ^15^, ^16^, ^17^, ^38^ and supported by the urolith analysis from 44 UUTU cases, of which 97.8% were CaOx. Additionally, median urine pH was 6.0 (IQR 5.5‐6.5) as would be expected in cats that have CaOx uroliths.^39^ Hypercalcemia was documented in 11.2% (total hypercalcemia) or 17.3% (ionized hypercalcemia; 17.3%) of UUTU cases in this study, and is a risk factor for CaOx urolith formation.^40^, ^41^ Additionally, cats with obstructive UUTU had significantly higher total and ionized calcium concentrations compared to cats with nonobstructive disease, suggesting this is not only a risk factor for UUTU formation, but might also be a contributory factor to the more aggressive obstructive phenotype.

Further limitations of this study must be considered. The retrospective design relied on the accurate recording of data in the clinical records, and there might have been confirmatory bias in the data available if it was more likely to be recorded based on previously identified risk factors (eg, diet being exclusively dry food).^42^ The dietary data included in the current study mainly described diet fed at the time of referral, but oftentimes it was not possible to ascertain how long these diets had been fed previously. Due to the high frequency of missing data for dietary information, an additional not‐reported category was created to allow inclusion in the second logistic regression model. However, the assumption that this data not being reported is independent of other variables in the model might not be safe. Cases were managed individually at each veterinarian's discretion and diagnostic tests were not standardized, therefore not all data were available for all cases. Importantly, there will have been diagnostic bias depending on the hospital service each cat originally presented to, for example, a cat seeing Dermatology might have been less likely to undergo abdominal imaging that a cat presenting to Internal Medicine. This might have contributed to some under‐reporting of UUTU in cats with uroliths but without relevant clinical signs, in particular the cats with nonobstructive uroliths. Although counts for UUTU cases per calendar year were available, it was not possible to report the proportion of cats seen per year diagnosed with UUTU, therefore only the 10‐year prevalence of UUTU has been reported for this hospital making it impossible to assess changes in prevalence over time during the study period. Additionally, our prevalence data should be considered in light of the average age of the cats studied in this cohort. Lastly, the group of cats studied was from a tertiary referral hospital and might be poorly representative of the general cohort of cats.

In conclusion, the prevalence of UUTU in the group of cats in this study was 4.6%. Independent risk factors for a diagnosis of UUTU were being female, being over 4 years of age, and being Burmese, Tonkinese, British shorthair, Ragdoll or Persian compared to non‐purebred cats. Within cats with uroliths, females, cats under 12 years of age and those with bilateral UUTU had increased odds for a diagnosis of obstructive UUTU. Cats with obstructive UUTU were younger than cats with nonobstructive UUTU, more likely to have ionized hypercalcemia and more likely to be eating exclusively dry food. Cats diagnosed with UUTU at a younger age therefore appear to have a more aggressive phenotype of this condition with a higher risk of ureterolithiasis and ureteral obstruction. Consideration should be given to monitoring younger cats with nephroliths closely for the development of ureteral obstruction. Further studies to enable identification of cats at risk of UUTU are warranted, to allow intervention before uroliths start to develop.

## CONFLICT OF INTEREST DECLARATION

Authors declare no conflict of interest.

## OFF‐LABEL ANTIMICROBIAL DECLARATION

Authors declare no off‐label use of antimicrobials.

## INSTITUTIONAL ANIMAL CARE AND USE COMMITTEE (IACUC) OR OTHER APPROVAL DECLARATION

Approved by the Royal Veterinary College Clinical Research Ethical Review Board (SR20181652).

## HUMAN ETHICS APPROVAL DECLARATION

Authors declare human ethics approval was not needed for this study.

## Supporting information


**Data S1.** Supporting Information.

## References

[1] Lulich JP , Berent AC , Adams LG , Westropp JL , Bartges JW , Osborne CA . ACVIM small animal consensus recommendations on the treatment and prevention of uroliths in dogs and cats. J Vet Intern Med. 2016;30:1564‐1574. 27611724 10.1111/jvim.14559 PMC5032870

[2] Kyles AE , Hardie EM , Wooden BG , et al. Management and outcome of cats with ureteral calculi: 153 cases (1984‐2002). J Am Vet Med Assoc. 2005;226:937‐944. 15786997 10.2460/javma.2005.226.937

[3] Berent AC , Weisse CW , Bagley DH , Lamb K . Use of a subcutaneous ureteral bypass device for treatment of benign ureteral obstruction in cats: 174 ureters in 134 cats (2009‐2015). J Am Vet Med Assoc. 2018;253:1309‐1327. 30398425 10.2460/javma.253.10.1309

[4] Kulendra NJ , Borgeat K , Syme H , Dirrig H , Halfacree Z . Survival and complications in cats treated with subcutaneous ureteral bypass. J Small Anim Pract. 2021;62:4‐11. 32926426 10.1111/jsap.13226

[5] Vrijsen E , Devriendt N , Mortier F , et al. Complications and survival after subcutaneous ureteral bypass device placement in 24 cats: a retrospective study (2016‐2019). J Feline Med Surg. 2021;23:759‐769. 33231515 10.1177/1098612X20975374 PMC10812192

[6] Lekcharoensuk C , Osborne CA , Lulich JP , et al. Trends in the frequency of calcium oxalate uroliths in the upper urinary tract of cats. J Am Anim Hosp Assoc. 2005;41:39‐46. 15634865 10.5326/0410039

[7] Ling GV , Ruby AL , Johnson DL , Thurmond M , Franti CE . Renal calculi in dogs and cats: prevalence, mineral type, breed, age, and gender interrelationships (1981‐1993). J Vet Intern Med. 1998;12:11‐21. 9503355 10.1111/j.1939-1676.1998.tb00491.x

[8] Kyles AE . Clinical, clinicopathologic, radiographic, and ultrasonographic abnormalities in cats with ureteral calculi: 163 cases (1984‐2002). J Am Vet Med Assoc. 2005;226:932‐936. 15786996 10.2460/javma.2005.226.932

[9] Houston DM . Canine and feline urolithiasis: examination of over 50 000 urolith submissions to the Canadian veterinary urolith centre from 1998 to 2008. Can Vet J. 2009;50:1263‐1268. 20190975 PMC2777289

[10] Houston DM , Vanstone NP , Moore AE , Weese HE , Weese JS . Evaluation of 21 426 feline bladder urolith submissions to the Canadian Veterinary Urolith Centre (1998‐2014). Can Vet J. 2016;57:196‐201. 26834273 PMC4713001

[11] Osborne CA , Lulich JP , Kruger JM , Ulrich LK , Koehler LA . Analysis of 451,891 canine uroliths, feline uroliths, and feline urethral plugs from 1981 to 2007: perspectives from the Minnesota Urolith Center. Vet Clin North Am Small Anim Pract. 2009;39:183‐197. 19038658 10.1016/j.cvsm.2008.09.011

[12] Cannon AB , Westropp JL , Ruby AL , Kass PH . Evaluation of trends in urolith composition in cats: 5,230 cases (1985‐2004). J Am Vet Med Assoc. 2007;231:570‐576. 17696857 10.2460/javma.231.4.570

[13] Burggraaf ND , Westgeest DB , Corbee RJ . Analysis of 7866 feline and canine uroliths submitted between 2014 and 2020 in the Netherlands. Res Vet Sci. 2021;137:86‐93. 33940351 10.1016/j.rvsc.2021.04.026

[14] Rogers KD . Composition of uroliths in small domestic animals in the United Kingdom. Vet J. 2010;188:228‐230. 20709586 10.1016/j.tvjl.2010.04.022

[15] Kopecny L , Palm CA , Segev G , Larsen JA , Westropp JL . Urolithiasis in cats: evaluation of trends in urolith composition and risk factors (2005‐2018). J Vet Intern Med. 2021;35:1397‐1405. 33955071 10.1111/jvim.16121 PMC8162610

[16] Mendoza‐López CI , Del‐Angel‐Caraza J , Aké‐Chiñas MA , et al. Epidemiology of feline urolithiasis in Mexico (2006–2017). JFMS Open Rep. 2019;5:205511691988569. 10.1177/2055116919885699 PMC6878601 31803489

[17] Butty EM , Labato MA . Subcutaneous ureteral bypass device placement with intraoperative ultrasound guidance, with or without microsurgical ureterotomy, in 24 cats. J Feline Med Surg. 2021;23:1183‐1191. 33754880 10.1177/1098612X211002014 PMC10812162

[18] Kennedy AJ , White JD . Feline ureteral obstruction: a case‐control study of risk factors (2016–2019). J Feline Med Surg. 2022;24:298‐303. 34076537 10.1177/1098612X211017461 PMC10812244

[19] Hsu H‐H , Ueno S , Miyakawa H , et al. Upper urolithiasis in cats with chronic kidney disease: prevalence and investigation of serum and urinary calcium concentrations. J Feline Med Surg. 2022;24:e70‐e75. 35471088 10.1177/1098612X221089856 PMC11104238

[20] Pimenta MM , Reche‐Júnior A , Freitas MF , Kogika MM , Hagiwara MK . Estudo da ocorrência de litíase renal e ureteral em gatos com doença renal crônica. Pesqui Vet Bras. 2014;34:555‐561.

[21] Ross SJ , Osborne CA , Lekcharoensuk C , Koehler LA , Polzin DJ . A case‐control study of the effects of nephrolithiasis in cats with chronic kidney disease. J Am Vet Med Assoc. 2007;230:1854‐1859. 17571990 10.2460/javma.230.12.1854

[22] www.cdc.gov/epiinfo/index.html.

[23] Broughton SE , O'Neill DG , Syme HM , et al. Ionized hypercalcemia in cats in a tertiary referral hospital: etiologies and association with urolithiasis. J Vet Intern Med. 2023;37:80‐91. 36645022 10.1111/jvim.16627 PMC9889682

[24] Wu YT , Hung WC , Huang PY , Tsai HJ , Wu CH , Lee YJ . Evaluation of and the prognostic factors for cats with big kidney‐little kidney syndrome. J Vet Intern Med. 2021;35:2787‐2796. 34655128 10.1111/jvim.16279 PMC8692197

[25] https://venomcoding.org/.

[26] Marz I , Wilkie LJ , Harrington N , et al. Familial cardiomyopathy in Norwegian Forest cats. J Feline Med Surg. 2015;17:681‐691. 25359788 10.1177/1098612X14553686 PMC11104058

[27] Osborne CA , Lulich JP , Thumchai R , et al. Feline urolithiasis. Etiology and pathophysiology. Vet Clin North Am Small Anim Pract. 1996;26:217‐232. 8711859

[28] Scales CD Jr , Smith AC , Hanley JM , Saigal CS . Prevalence of kidney stones in the United States. Eur Urol. 2012;62:160‐165. 22498635 10.1016/j.eururo.2012.03.052 PMC3362665

[29] Ferraro PM , Taylor EN , Curhan GC . Factors associated with sex differences in the risk of kidney stones. Nephrol Dial Transplant. 2023;38:177‐183. 35138394 10.1093/ndt/gfac037 PMC9869853

[30] Khan SR , Pearle MS , Robertson WG , et al. Correction: kidney stones. Nat Rev Dis Primers. 2017;3:17001. 28079115 10.1038/nrdp.2017.1

[31] Peerapen P , Thongboonkerd V . Protective cellular mechanism of estrogen against kidney stone formation: a proteomics approach and functional validation. Proteomics. 2019;19:1900095. 10.1002/pmic.201900095 31475403

[32] Kienzle E , Moik K . A pilot study of the body weight of pure‐bred client‐owned adult cats. Br J Nutr. 2011;106(Suppl 1):S113‐S115. 22005404 10.1017/S0007114511001802

[33] Merenda MEZ , Sato J , Scheibel S , et al. Growth curve and energy intake in male and female cats. Top Companion Anim Med. 2021;44:100518. 33549804 10.1016/j.tcam.2021.100518

[34] Tang PK , Jepson RE , Chang YM , Geddes RF , Hopkinson M , Elliott J . Risk factors and implications associated with renal mineralization in chronic kidney disease in cats. J Vet Intern Med. 2022;36:634‐646. 35043997 10.1111/jvim.16363 PMC8965253

[35] Rule AD , Krambeck AE , Lieske JC . Chronic kidney disease in kidney stone formers. Clin J Am Soc Nephrol. 2011;6:2069‐2075. 21784825 10.2215/CJN.10651110 PMC3156433

[36] Shang W , Li L , Ren Y , et al. History of kidney stones and risk of chronic kidney disease: a meta‐analysis. PeerJ. 2017;5:e2907. 28149686 10.7717/peerj.2907 PMC5267565

[37] Cléroux A , Alexander K , Beauchamp G , Dunn M . Evaluation for association between urolithiasis and chronic kidney disease in cats. J Am Vet Med Assoc. 2017;250:770‐774. 28306489 10.2460/javma.250.7.770

[38] Gerber B , Brandenberger‐Schenk F , Rothenanger E , Müller C . Uroliths of cats in Switzerland from 2002 to 2009. Schweiz Arch Tierheilkd. 2016;158:711‐716. 27707684 10.17236/sat00089

[39] Bartges JW . Feline calcium oxalate urolithiasis: risk factors and rational treatment approaches. J Feline Med Surg. 2016;18:712‐722. 27562981 10.1177/1098612X16660442 PMC11148900

[40] Midkiff AM . Idiopathic hypercalcemia in cats. J Vet Intern Med. 2000;14:619‐626. 11110384 10.1892/0891-6640(2000)014<0619:ihic>2.3.co;2

[41] Savary KC . Hypercalcemia in cats: a retrospective study of 71 cases (1991‐1997). J Vet Intern Med. 2000;14:184‐189. 10772491 10.1892/0891-6640(2000)014<0184:hicars>2.3.co;2

[42] McKenzie BA . Veterinary clinical decision‐making: cognitive biases, external constraints, and strategies for improvement. J Am Vet Med Assoc. 2014;244:271‐276. 24432957 10.2460/javma.244.3.271

